# Effectiveness of a Brief Online Mindfulness-Based Intervention for University Students

**DOI:** 10.1007/s12671-023-02128-1

**Published:** 2023-05-02

**Authors:** Sabrina Fagioli, Susanna Pallini, Stefano Mastandrea, Barbara Barcaccia

**Affiliations:** 1 Department of Education, Roma Tre University, Via Di Castro Pretorio N.20, 00185 Rome, Italy; 2 Department of Psychology, Sapienza University of Rome, Via Dei Marsi N.78, 00185 Rome, Italy; 3 Associazione Di Psicologia Cognitiva APC and Scuola Di Psicoterapia Cognitiva Srl SPC, Viale Castro Pretorio N. 116, 00185 Rome, Italy

**Keywords:** Online mindfulness intervention, Online university class, Attention related to interoceptive awareness, Sense of community, Academic self-efficacy

## Abstract

**Objectives:**

The COVID-19 pandemic resulted in a dramatic increase in Web-based education, lacking face-to-face student–teacher and student–student interaction, and consequently impairing students’ sense of belonging to a community, interoceptive awareness, and academic self-efficacy. This study examined how a brief mindfulness-based intervention in an online university course can be effective in enhancing attention resources, developing a stronger sense of academic self-efficacy, and improving the sense of belonging to a community, which represent critical factors affecting students’ participation in online and blended courses.

**Method:**

Four-hundred and eighty-six participants (*M*_*age*_ 22.88) completed a battery of measures at pre- and post-treatment. One class (experimental group) participated in a brief online mindfulness-based intervention (42%), whereas the other one (control group) did not take part in the intervention (58%). The intervention included breathing meditation at the beginning of class, sharing of experiences, mini-lectures on mindfulness, and daily practice, and lasted for 28 consecutive days.

**Results:**

Participants in the experimental group when compared to controls showed a significant increase in the feeling of influencing the course activities (*F* = 9.628; *p < *0.005), in the self-regulation of attention (*F* = 19.133; *p < *0.001), in academic self-efficacy (*F* = 9.220; *p < *0.005), and, particularly, in their self-efficacy in regulating learning (*F* = 12.942; *p < *0.001). The students’ adherence to the assigned practice could partially explain the effectiveness of the intervention.

**Conclusions:**

This study offers useful clues about the effectiveness of mindfulness interventions in the classroom in enhancing sense of belonging to a community, attention grounded in bodily sensations, and academic self-efficacy.

**Preregistration:**

This study is not preregistered.

The COVID-19 pandemic has had a significant impact on education, leading to a decrease in face-to-face interaction and an increase in Web-based education (Daniel, 2020). This shift has resulted in challenges for students and faculty members, including cancelled practical activities, inadequate support services for online teaching, and negative effects on students’ sense of belonging to a community, interoceptive awareness, and academic self-efficacy (Al-Kumaim et al., 2021; Cranfield et al., 2021).

First, the nationwide lockdowns and the consequent social distancing and extensive use of a virtual learning environment have reduced social opportunities and contacts, increased feelings of loneliness, and impaired the sense of community and relatedness (Butz & Stupnisky, 2016; Oliveira et al., 2022; Okruszek et al., 2020; Ritter et al., 2010). Second, online learning has been found to affect attention due to distracting household activities, impaired interpersonal communication, and relationship challenges (Auccahuasi, 2021; Barbu et al., (2022; Pallini et al., 2019a, 2019b). Indeed, students live in two places simultaneously: their home and the learning environment. Furthermore, Web-based communication is limited to only sight and hearing, and does not allow for the use of all five senses, whereas attention is biologically related to proprioceptive/interoceptive body awareness, which is defined as the conscious perception of internal signals, including heartbeat and breathing (Craig, 2002). Body awareness is characterized by the sense of “Embodiment,” that is, the feeling of being self-grounded in experiencing physical sensations in the present moment, entailing both “Thinking about the body” and “Presence in the body” (Mehling et al., 2012). Body awareness can be enhanced by regulating attention and focussing on immediately experienced feelings. Finally, isolation and challenged interoceptive awareness could influence students’ academic self-efficacy, i.e., their “capabilities to organize and execute courses of action required to produce given attainments” (Bandura, 1997, p. 3). All these specific aspects, such as the sense of belonging to a community, interoceptive awareness, and academic self-efficacy, represent critical factors for students’ participation in online and blended courses, and for the subjective perception of learning (Rovai & Wighting, 2005; Vayre & Vonthron, 2017; Vecchio et al., 2022).

Mindfulness-based interventions (MBIs) have been found to be beneficial in improving students’ psychological health, as well as positive affect, emotional experience, and learning-related processes, such as academic performance and knowledge retention (Bennett et al., 2018; Calma-Birling & Gurung, 2017; Critchley & Garfinkel, 2017; Galante et al., 2018, 2021; Lin & Mai, 2018; Ramsburg & Youmans, 2014; VanKuiken et al., 2017; Vorontsova-Wenger et al., 2022; Wiens, 2005; Yamada & Victor, 2012). Mindfulness is the non-judgmental acceptance of one’s thoughts, feelings, and sensations as they arise, moment by moment (Jennings & Jennings, 2013; Kabat-Zinn, 2003). It entails being aware of and paying attention to present-moment experiences with an accepting, non-judgmental, and curious attitude (Regehr et al., 2013; Segal et al., 2013). This practice has been found to be positively associated with well-being (Barcaccia et al., 2020, 2022; Ioverno et al., 2022; Medvedev et al., 2021). Specifically, in-person MBIs have been associated with reduced loneliness, decreased mind-wandering, and the occurrence of distracting thoughts which can increase interoceptive awareness (Bornemann et al., 2015; Farb et al., 2013, 2015; Gibson, 2019; Hanley et al., 2017; Loucks et al., 2021; Miller et al., 2019; Mrazek et al., 2013; VanKuiken et al., 2017; Zhang et al., 2018). By focussing attention on interoception of breathing and body, mindfulness practice enhances body awareness and develops interoceptive awareness (Farb et al., 2015). Students who take part in MBIs, when distracted, become more capable to be aware of distractions, so can regain their focus and improve classroom attention and awareness (Michael et al., 2020). The study by VanKuiken et al. (2017) showed that students who benefited from an in-person short intervention in class found the experience calming, allowing them to focus, set aside distractions, and feel a sense of community, although these findings were exclusively based on qualitative evaluations.

Finally, mindfulness has been found to be associated to academic self-efficacy, increasing perceptual clarity, facilitating accurate self-perception, counteracting negative self-bias, and promoting adaptive perception of the self (Hanley et al., 2017). Taylor et al. (2020) showed the effectiveness for self-efficacy of an 8-week classroom MBI encompassing meditations, reflective journaling, and group discussions. Vidic and Cherup (2019) also evidenced the benefits of a 7-week MBI for undergraduate students’ general self-efficacy and stress levels. Academic self-efficacy, particularly students’ beliefs in their efficacy to regulate their own learning and academic attainments, has been found to be related to the sense of community, mindful attention awareness, and scholastic achievement by promoting high academic aspirations and reducing vulnerability to feelings of futility and depression (Bandura et al., 1996; Uzdil & Günaydın, 2022; Zysberg & Schwabsky, 2021).

In-person MBIs can be effectively implemented into college courses as they are usually shorter than more traditional interventions, and thus easier to incorporate into class schedules. Typically, they involve mindfulness meditation practices in the classroom and at home (Calma-Birling & Gurung, 2017; Lin & Mai, 2018; Michael et al., 2020; Miller et al., 2019; Taylor et al., 2020; VanKuiken et al., 2017; Yamada & Victor, 2012). Many MBIs for university students include the use of audio files or books (Bennett et al., 2018; Lloyd et al., 2018; Taylor et al., 2020).

In order to better face the COVID-19 pandemic, several MBIs for university students have been adapted to the online format, and varied for frequency, training model, teaching materials, and outcomes pursued (Currie, 2020). However, only a handful of studies investigated the effectiveness of online MBIs to support students during COVID-19. These studies have shown the benefits of online MBIs during regular class hours for various aspects of psychological adjustment, but not for the sense of belonging to a community, interoceptive awareness, and academic self-efficacy. This is surprising, given that these aspects have been negatively affected by online learning and represent critical factors for students’ participation in online and blended courses. Consequently, further investigation would be useful to explore these effects (Rovai & Wighting, 2005; Vayre & Vonthron, 2017; Vecchio et al., 2022).

Considering the above-reviewed evidence, we aimed to examine whether a brief online MBI during regular university class hours in the context of remote emergency learning was effective in increasing interoceptive awareness, developing a stronger sense of academic self-efficacy, and improving the sense of belonging to a community (from now on *sense of community*).

Following the results of in-person MBIs (Loucks et al., 2021; Taylor et al., 2020; Vidic & Cherup, 2019; Zhang et al., 2018;), we predicted that a brief online MBI encompassing breathing meditation at the beginning of class, sharing of experiences, mini-lectures on mindfulness, and daily practice, would be associated in the experimental, but not in the control group, with an increase of university students’ (1) sense of community, (2) interoceptive awareness, (3) academic self-efficacy, and specifically, the belief of being capable of organizing and planning activities to achieve university goals. To this end, using cluster randomization, one class was assigned to the experimental group (with mindfulness intervention) and the other to the control group (without intervention). Our intervention was implemented online during regular class-hours and included not only formal practices (namely, breathing meditation), but also a subsequent phase of shared experiences connected to the mindfulness practice. Students could share their experience of meditation, and the instructor took the opportunity of illustrating the core elements of mindfulness (mini-lectures). The intervention also entailed daily practice at home.

## Method

### Participants

The study took place during the spring semester of 2022, from April 20 to May 11. The sample for this study was selected using a cluster randomization procedure, in which pre-existing groups (classes) were randomly assigned to receive the mindfulness intervention. Participants were students enrolled in two undergraduate classes in General Psychology in a bachelor’s degree in Education at X (blinded for review). The two classes had identical learning goals, materials, contents, and assignments, as outlined in their syllabi. The experimental group consisted of students from one class, while the control group consisted of students from the other class.

The recruited participants were 672. Of these, 186 were excluded because they had withdrawn from class. It is typical for students to change their class schedule in the first few weeks of any given semester. The final sample consisted of 486 participants: 205 (42%) in the experimental group (class 1) and 281 (58%) in the control group (class 2). Specifically, the experimental group took part in the brief online MBI, whereas the control group was composed of non-meditating participants. The mean age of the participants was 22.88 (range: 19–60; *SD* = 6.39) and the sample consisted of 14 male (3%) and 472 (97%) female students.

### Procedure

Two professors of General Psychology—the first and third authors of this article—gave lectures in the two classes, which were composed of a similar number of students. Lectures were conducted at the same time, in comparable virtual classrooms, and used a similar class format including group discussions. Classes met for 2 hr twice a week for 12 consecutive weeks. The mindfulness intervention was delivered by the last author of this article, who is a clinical psychologist with extensive experience teaching mindfulness meditation and advanced training in delivering MBIs in both clinical and non-clinical settings, including formal MBSR instruction. Students in the control group did not have any lectures with this instructor; she only delivered the MBI in the experimental group. In the control group, the course proceeded as usual with the same content of the experimental group, but no mindfulness intervention was provided. Both groups spent the same amount of time in the virtual class, so there was no additional time dedicated to the mindfulness intervention. Instead, the lecturer in the control group conducted a break session, in order to balance the time spent in the learning process between the two groups. The data questionnaires were collected at two specific points in time: 1 week before the start of the mindfulness training and 1 week after its completion. The course, the self-administered questionnaires, and the mindfulness intervention were delivered online on Web platforms.

#### Consent

This study was part of a larger survey including additional variables on the effectiveness of a mindfulness program for the psychological well-being of university students. Prior to the study, participants were provided with the information sheet including a general overview of the study and procedures. All participants provided written informed consent and completed a battery of instruments online (pre- and post-treatment). No adverse experiences related to the study intervention were reported by the participants. All the students who took part in the study received the same grade incentives (both the experimental and the control groups).

#### Mindfulness Intervention

The practice proposed in the experimental group was derived from the principles of mindfulness-based stress reduction (MBSR; Kabat-Zinn, 1990) and consisted of a brief introductory explanation followed by guided sitting meditation. Before starting the meditation, participants were asked to sit on a chair. The instructions provided before the beginning of the breathing meditation emphasized the physical posture and mental strategies of focussed-attention meditation (Kabat Zinn, 2013; Kabat-Zinn, 2016). The instructions provided for the breathing meditation emphasized the following: (a) sitting in an upright posture with legs uncrossed and eyes closed. If they felt uncomfortable in keeping their eyes closed, they could keep them open and lower their gaze; (b) using the breath as an anchor for attention during meditation; (c) allowing the mind to rest naturally rather than trying to suppress the occurrence of thoughts.

In the course of the sitting meditation, students were instructed to gently re-focus on breathing every time their minds started wandering, and to pay attention to their internal and external moment-by-moment experiences with openness, curiosity, and acceptance. Immediately following the guided meditation session, participants were invited to share their experiences with the class and received feedback from the mindfulness instructor, who included in the comments to the students’ experiences the illustration of the main pillars of mindfulness: Non-judging, Patience, Beginner’s Mind, Trust, Non-Striving, Acceptance, Letting Go (Kabat-Zinn, 2013). At the end of each guided meditation, the regular lecture was delivered by the General Psychology professor. The duration of the mindfulness guided intervention (i.e., meditation + sharing experience + mini-lecture) lasted about 30 min. Participants were given an audio file of the 10-min breathing meditation and were asked to practice it daily (including the same day as the in-class session).

Both the experimental and the control groups proceeded as usual with the same content for the course and spent an equal amount of time in the virtual class, so no extra time was allocated for the mindfulness intervention in the experimental group. To balance the time spent in the learning process between the two groups, the professor of General Psychology in the control group conducted a break session during the middle of the class. In both classes, students were asked to disengage from social media at the beginning of each class session.

Over a period of 28 days, participants in the experimental group engaged in four in-class guided meditation sessions (one per week) and a daily mindfulness practice: for 4 consecutive weeks, the last author of this study led a 10-min breathing meditation at the beginning of each Tuesday’s class as part of the in-class guided meditation sessions. No intervention was provided in the control group.

At the end of each day, students in the experimental group completed a brief survey (a Google form link sent every day by email) to evaluate their adherence to the daily mindfulness practice. The whole intervention duration (guided breathing meditation in-class + daily practice at home) was 28 consecutive days.

### Measures

#### The Multidimensional Assessment of Interoceptive Awareness

The Multidimensional Assessment of Interoceptive Awareness (MAIA; Mehling et al., 2012) is a 32-item self-report measure on a 6-point Likert scale. The Italian version by Calì et al. (2015) includes 8 subscales: (1) noticing (*I notice where in my body I am comfortable*, 4 items); (2) not-distracting *(I distract myself from sensations of discomfort*, 3 items); (3) not-worrying (*I start to worry that something is wrong if I feel any discomfort*, 3 items); (4) attention regulation (*I am able to consciously focus on my body as a whole*, 7 items); (5) emotional awareness (*When something is wrong in my life, I can feel it in my body*, 5 items); (6) self-regulation (*I can use my breath to reduce tension*, 4 items); (7) body listening (*I listen to my body to inform me about what to do*, 3 items); (8) trusting (*I trust my body sensations*, 3 items). Items ranged from 0 (*never*) to 5 (*always*) along a Likert scale, with higher scores indicating better interoceptive awareness. The MAIA subscales have demonstrated acceptable psychometric properties (Brown et al., 2017; Machorrinho et al., 2019; Mehling et al., 2012). In our sample, the subscales in T1 and T2 have shown a good internal consistency (Cronbach’s alpha range = 0.66–0.72, and McDonald’s omega = 0.89–0.89).

#### The Scale of Sense-of-Community in University Online Courses (SSC-CUO) McMillan & Chavis, 1986)

The Italian version of the Scale of Sense of Community in University Online Courses (SSC-CUO) by Balboni et al. (2018), based on the model by McMillan and Chavis (1986), includes 36 items which have been distinguished in three subscales: (1) membership, that measures the quantity/quality of interaction between the members of an online community (*If I don’t understand something, I can rely on the support of the other students attending the course*, 19 items); (2) influence, that is the subjective feeling of influencing the course activities (*My participation is relevant to the overall success of the course*, six items); (3) fulfilment of needs, the fulfilment of students’ learning needs (*Attending the course is a source of personal satisfaction*, 11 items). Items ranged from 0 (strongly disagree) to 4 (strongly agree) along a Likert scale, with higher scores indicating higher sense-of-community. The SSC-CUO subscales have demonstrated acceptable psychometric properties (Balboni et al., 2018). In our sample, the subscales have shown a good internal consistency, ranging from (T1–T2) Cronbach’s alpha = 0.77–0.74, Mc Donald’s omega = 0.77–0.75.

#### Academic Perceived Self-Efficacy Scale

The Academic Perceived Self-Efficacy Scale (Bandura et al., 1996; Burgalassi et al., 2016; Pastorelli et al., 2001) includes 10 items related to two broad domains of self-efficacy beliefs. The first domain refers to the perceived capacity in regulating academic learning activities (e.g., *How well can you take good notes during the lectures*). The second domain concerns the capability in organizing and planning the study (e.g., *How well can you study when there are other interesting things to do?*). Items ranged on a Likert scale from 1 (*cannot do at all*) to 5 (*highly certain can do*). The psychometric properties of this scale have been validated in large samples of Italian respondents as well as in cross-cultural comparisons (Pastorelli et al., 2001). In our sample, the subscales have shown a high internal consistency, ranging from (T1–T2) Cronbach’s alpha = 0.91–0.90, McDonald’s omega = 0.92–0.91.

#### Adherence to the Daily Mindfulness Meditation Practice

In order to assess mindfulness practice dose–response effects, we assessed participants’ practice throughout the 4-week intervention. Various aspects of adherence to the daily practice were explored through four questions: participants were sent each night a reminder with a link to the survey. For the purpose of the present study, we only analyzed the responses to the first question: *Did you practice today breathing meditation with the provided audio recording?* The response format was dichotomous (yes/no).

### Data Analyses

Statistical analyses were performed using IBM SPSS Statistics version 26. Group difference on the gender variable was assessed with the* χ*^2^ test. Independent *t*-tests were used to assess differences between groups in terms of age.

To explore differences on diverse variables between participants who received mindfulness training (experimental group) and participants who did not (control group), we performed multiple mixed models of univariate analysis of variance (ANOVA) (Huberty & Morris, 1989) considering Time (pre- vs post-intervention) as within-subjects, and Group (experimental vs control) as between-subjects factors. Post hoc analysis of simple interaction effects was performed using pairwise comparisons on the Bonferroni-adjusted estimated marginal means.

Responses of the brief daily survey about the adherence to the daily mindfulness practice were analyzed through analysis of covariance (ANCOVA). Univariate ANCOVAs were conducted to examine the change score (pre- vs post-intervention) on the dependent variables using adherence to the practice as covariate. In particular, the “adherence score” was computed for each subject dividing the number of days practicing mindfulness by the total duration in days of the intervention (28 days). In the experimental group, 194/205 participants completed the daily survey to register adherence at least once during the intervention period. The level of statistical significance used for interpreting univariate analyses was defined as *p* < 0.05 (Bonferroni adjusted, see below). All numerical outputs are rounded to two decimal places, except for *p*-values that have been adjusted by the Bonferroni correction, which are rounded to three decimal places to show their fit with the correction.

## Results

### Differences at Baseline Between Experimental and Control Groups

Participants of the experimental and control group did not differ regarding age (mean age (*SD*): 22.76 (6.47) vs 22.97 (6.35), respectively; *t*(484) =  − 0.36; Hedges’ *g* = 6.41; *p* = 0.72). Means and standard deviations regarding all the explored variables for both the experimental and the control group at pre- and post-intervention are shown in Table 1.

#### Academic Self-efficacy

Students in the control group obtained higher scores at pre-intervention compared to the experimental group on self-efficacy in regulating university learning (3.19 vs 2.99; *p* < 0.05; Hedges’ *g* =  − 0.30) and academic self-efficacy (3.18 vs 2.99; *p* < 0.001; Hedges’ *g* =  − 0.32). Independent post hoc comparisons revealed that pre-intervention scores on these scales significantly differed between groups.

#### Multidimensional Assessment of Interoceptive Awareness

Independent post hoc comparisons also showed that participants of the control group obtained higher scores at pre-intervention compared to the experimental group on self-regulation (2.84 vs 2.56; *p* < 0.001; Hedges’ *g* =  − 0.33) and trusting (3.37 vs 3.12; *p* < 0.05; Hedges’ *g* =  − 0.23), but not on attention regulation.

#### Sense of Community for Online Course

Participants in the control group showed higher scores on the Influence subscale of the Sense of Community scale at pre-intervention compared to the experimental group (2.30 vs 2.75; *p* < 0.001; Hedges’ *g* =  − 0.34). No other effects reached significance or survived Bonferroni correction.

### Differences at Post-intervention Between Experimental and Control Groups

#### Academic Self-efficacy

**Fig. 1:**
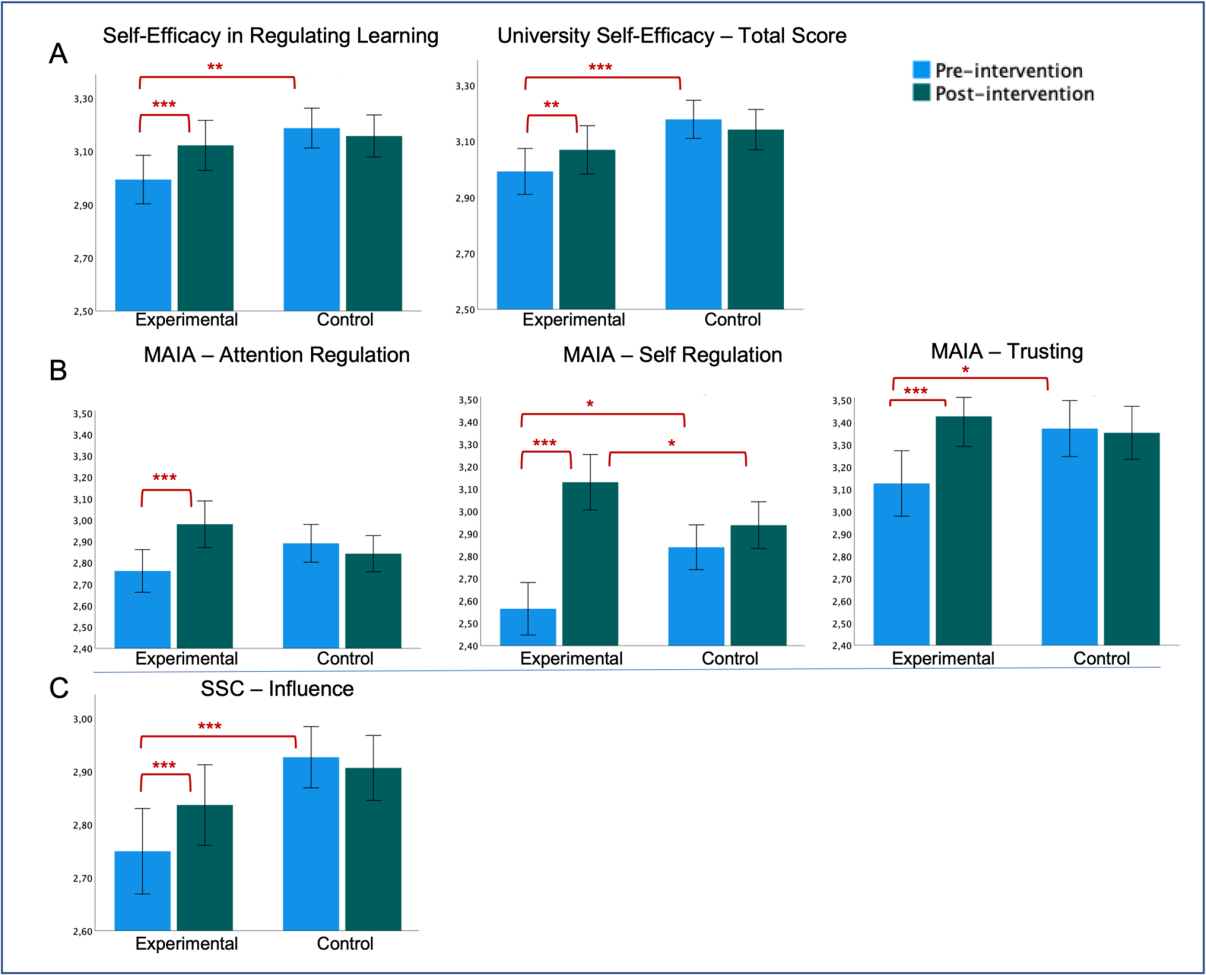
Estimated Marginal Means (EMM) for academic self-efficacy (panel **A**), Multidimensional Assessment of Interoceptive Awareness (panel **B**), and sense of community for online course (panel **C**) on pre-training and post-training assessment, for the experimental and the control groups. Black bars indicate 95% confidence interval of the EEM. Red lines between bars represent significant pairwise comparisons. *p < 0.05; **p < 0.01; ***p < 0.001: *p*-values for differences between conditions

Results of the ANOVAs performed on the academic self-efficacy scores obtained after adjusting *p*-values with Bonferroni procedure (i.e., 0.05/12 = 0.0042) revealed significant effects of time by group interaction for both self-efficacy in regulating university learning (*F*_(1.484)_ = 12.94; *p* < 0.001; *η*^2^ = 0.03) and self-efficacy total score (*F*_(1.484)_ = 9.22; *p* = 0.003; *η*^2^ = 0.02). Paired comparisons showed that participants who attended the mindfulness intervention increased self-efficacy in regulating university learning (*M* = 2.99 vs 3.12 for pre- and post-training, respectively, *p* < 0.001; Hedges’ *g* =  − 0.27; Fig. 1, panel A) than participants in the control group (*M* = 3.19 vs 3.16 for pre- and post-intervention, respectively, *p* = 0.30; Hedges’ *g* =  − 0.06). Moreover, participants who attended the mindfulness intervention reported a higher total score on academic self-efficacy (2.99 vs 3.07 for pre- and post-intervention, respectively, *p* < 0.01; Hedges’ *g* =  − 0.19; Fig. 1, Panel A) than participants who did not (3.18 vs 3.14 for pre- and post-intervention, respectively, *p* = 0.13; Hedges’ *g* =  − 0.09).

#### Multidimensional Assessment of Interoceptive Awareness

After adjusting for multiple comparisons (i.e., 0.05/32 = 0.0016), the ANOVAs performed on the multidimensional assessment of interoceptive awareness scale showed that participants of both groups had increased scores on emotional awareness (*F*_(1,484)_ = 17.09; *p* < 0.001; *η*^2^ = 0.03), self-regulation (*F*_(1,484)_ = 75.44; *p* < 0.001; *η*^2^ = 0.13), body listening (*F*_(1,484)_ = 24.26; *p* < 0.001; *η*^2^ = 0.05), and trusting (*F*_(1,484)_ = 12.37; *p* < 0.001; *η*^2^ = 0.02). Furthermore, significant Group by Time interaction effects were found for attention regulation (*F*_(1,484)_ = 19.13; *p* < 0.001; *η*^2^ = 0.04), self-regulation (*F*_(1,484)_ = 37.21; *p* < 0.001; *η*^2^ = 0.07), and trusting (*F*_(1,484)_ = 15.93; *p* < 0.001*; η*^2^ = 0.03). After taking part in the mindfulness training, participants in the experimental group were more capable of paying attention to body sensations (*M* = 2.76 vs 2.98, for pre- and post-intervention, respectively, *p* < 0.001; Hedges’ *g* =  − 0.30; Fig. 1, panel B), regulating distress by paying attention to body sensations (*M* = 2.56 vs 3.12, for pre- and post-intervention, respectively, *p* < 0.001; Hedges’ *g* =  − 0.62; Fig. 1, Panel B) and trusting their own body sensations (*M* = 3.12 vs 3.43, for pre- and post-intervention, respectively, *p* < 0.001; Hedges’ *g* =  − 0.31; Fig. 1, Panel B).

#### Sense of Community for Online Course

Results of ANOVA models obtained after adjusting *p*-values for multiple comparisons (e.g., 0.05/12 = 0.0042) showed a main effect of time on membership scores (*F*_(1,484)_ = 12.477; *p* < 0.001; *η*^2^ = 0.02), highlighting that scores on this dimension decreased over time for both groups (*M* = 3.24 vs 3.19). Furthermore, a significant interaction effect between time and group was revealed for the influence dimension (*F*_(1,484)_ = 9.63; *p* = 0.002; *η*^2^ = 0.02), with participants of the experimental group showing a significant increase in the perception of influence of the individuals and the community at post-intervention (*M* = 2.75 vs 2.84 for pre- and post-training, respectively, *p* < 0.001; Hedges’ *g* =  − 0.24; Fig. 1) compared to control participants (*M* = 2.93 vs 2.91 for pre- and post-training, respectively, *p* = 0.37; Hedges’ *g* = 0.05; Fig. 1).

### Adherence to the Meditation Practice

During the 4-week intervention period, participants in the experimental group meditated for an average of 17 days (range: 0–27, *SD* = 7.99; median = 19; mode = 22). This did not include the days when they participated in the online in-class breathing meditation. ANCOVAs conducted on the academic self-efficacy scale revealed a significant interaction effect of the time by Covariate term for self-efficacy in regulating university learning (*F*_(1,192)_ = 13.40; *p* < 0.001; *η*^2^ = 0.95) and self-efficacy total score (*F*_(1,192)_ = 7.20; *p* = 0.01; *η*^2^ = 0.76). This result indicates that the academic self-efficacy score has been incremented by the mindfulness-based intervention.

**Fig. 2:**
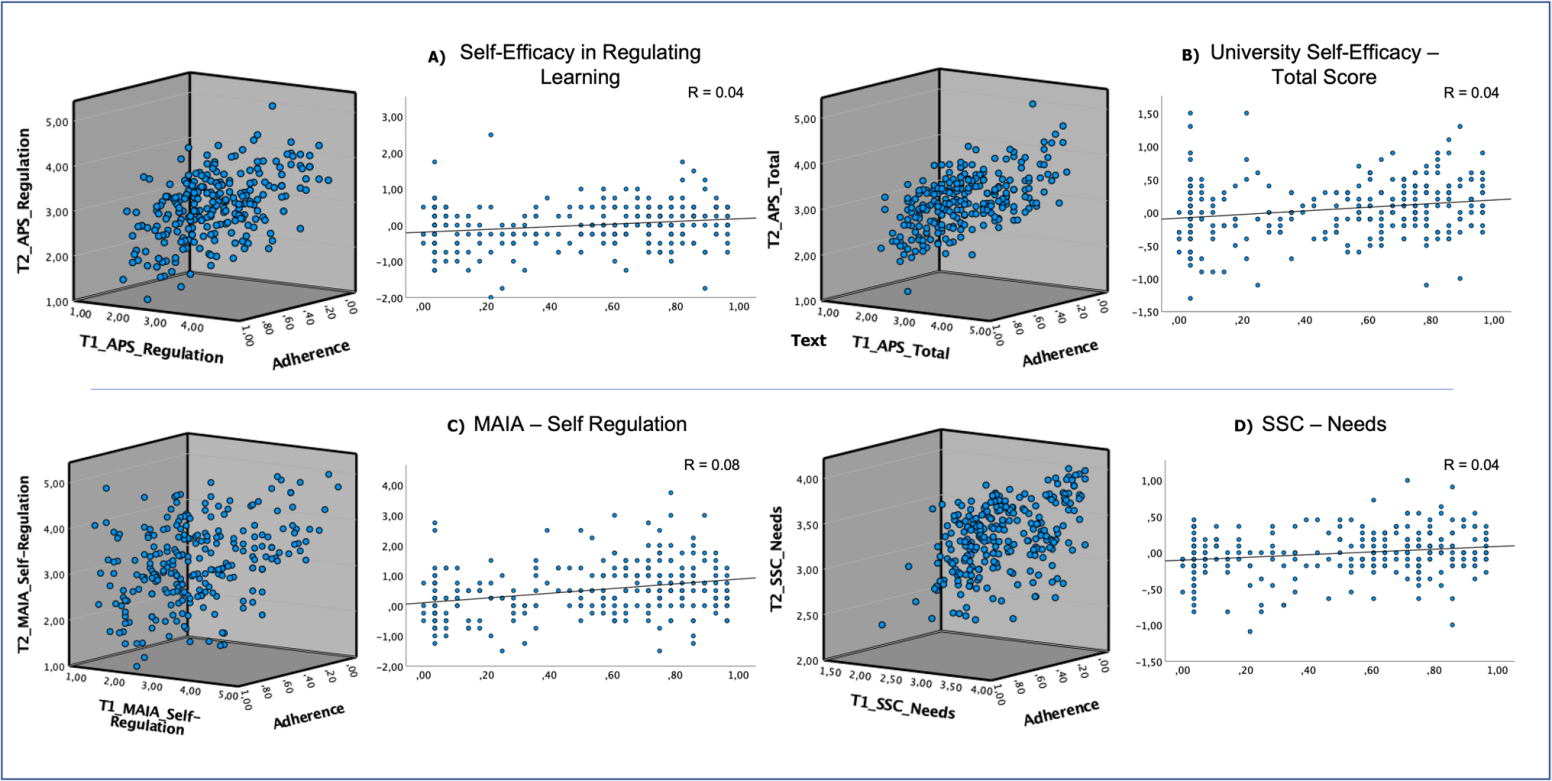
Modulation on academic self-efficacy (self-efficacy in regulating learning and Total Score, top panel), Multidimensional Assessment of Interoceptive Awareness (self-regulation, bottom panel–left), and sense of community in university online courses (needs, bottom panel–right) by the adherence to the daily practice. For these sub-scales, the success of the in-class online mindfulness intervention was partly modulated by the adherence to the daily practice. In particular, for each subscale, the 3-D scatter plot shows the positive linear relationship between the score at pre-training (T1, x-axis), the score at post-training (T2, y-axis), and the mean value of adherence for each participant (z-axis). Similarly, the 2-D scatter plot shows the positive linear relationship between the mean adherence score and the gain score of the intervention (i.e., post-training score – pre-training score)

Analogously, we found that adhering to the mindfulness daily practice modulated—slightly but significantly—the increase of MAIA self-regulation (*F*_(1,192)_ = 13.22; *p* < 0.001; *η*^2^ = 0.95) and SSC fulfilment of needs scores (*F*_(1,192)_ = 8.95; *p* = 0.01; *η*^2^ = 0.84) from pre- to post-intervention assessment. Other significant effects did not survive the Bonferroni correction. Therefore, meditating on a daily basis has been shown to ameliorate the MAIA self-regulation (Fig. 2, C) and increase the self-efficacy in regulating learning (Fig. 2, A). For these sub-scales, the effectiveness of the in-class online mindfulness intervention was partly modulated by the adherence to the daily practice. In particular, for each subscale, the 3-D scatter plot shows the positive linear relationship between scores at pre-intervention (T1, x-axis), scores at post-intervention (T2, y-axis), and the mean value of adherence for each participant (z-axis). Similarly, the 2-D scatter plot shows the positive linear relationship between the mean adherence score and the gain score of the intervention (i.e., post-intervention score – pre-intervention score).

In summary, a significant variable accounting for the benefits of our MBI is its amount, i.e., the dose–response relationship, which is the association between the amount of exposure (dose) to the mindfulness meditation practice and the resulting changes (response): the effects of the intervention have also been modulated by the amount of mindfulness practice that the participants completed in their extra-academic time (Fig. 2).

## Discussion

This study investigated the effects of a mindfulness intervention in an on-line university class on the sense of community, on the interoceptive awareness in an online environment, and on academic self-efficacy. To this end, an experimental group practicing mindfulness was compared to a control group without mindfulness intervention. Several aspects of the methodological design, particularly the presence of a control group, support the hypothesis that the observed experimental group’s improvements in the explored dimensions could be due to the online brief MBI.

Regarding the sense of community within the online course, participants of the experimental group showed a significant increase in the perception of influence of the individuals and of the community at post-treatment, when compared to controls. Previous research has shown that practicing mindfulness in a group setting can increase a sense of belonging among participants (Russo, 2019). Mindfulness interventions have been found to counteract the feelings of loneliness and isolation resulting from the nationwide lockdowns, social distancing, and transition from in-person to online learning (Oliveira et al., 2022; Okruszek et al., 2020; Ritter et al., 2010).

In our study, the sense of community might have been enhanced by the discussion following the in-class breathing meditation, centered on the disclosing and sharing of the students’ personal experiences. Mindfulness orients individuals towards clearly seeing internal and external stimuli and events as they are, increasing bodily calm and cultivating an attitude of openness and acceptance towards oneself and the others. Breathing meditation enhances receptivity, leading to attunement to the humanity of others (Kramer et al., 2023). Our findings suggest that this could also result from an online brief MBI.

Regarding the interoceptive awareness, only participants of the experimental group improved their capability to regulate attention and psychological distress by both paying attention to and trusting bodily sensations, thus protecting students from possible dissociative phenomena (Mehling et al., 2012) due to the contemporary presence in virtual and real environments. Focusing on physical sensations arising from one’s body or resulting from contact with a chair can strengthen the sense of connectedness to oneself. Our study indicates that promoting mindfulness among university students can enhance connectedness to oneself and others through a combination of individual and group practices, which can in turn facilitate the learning process (Russo, 2019).

Finally, regarding the perception of academic self-efficacy, participants in the experimental group increased confidence in their capacity of regulating learning from pre- to post-treatment and reported higher total scores on academic self-efficacy compared to controls, counteracting the effects of isolation and impaired attentive processes in an online learning environment (Heo et al., 2021). The students’ adherence to the assigned practice during the week could partially explain the success of the intervention: one of the most important posited features explaining the positive effects of MBIs is participants’ engagement in regular home practice (Lloyd et al., 2018).

These results corroborate previous similar evidence stemming from studies investigating the effectiveness of in-person MBIs for loneliness and self-efficacy, and for attention-related processes, contributing to demonstrate the effectiveness of online MBIs for these variables, which was not investigated before (Bishop et al., 2004; Bornemann et al., 2015; Dawson et al., 2020; Farb et al., 2013, 2015; Gibson, 2019; Hanley et al., 2017; Loucks et al., 2021; Miller et al., 2019; Mrazek et al., 2013; Taylor et al., 2020; VanKuiken et al., 2017; Vidic & Cherup, 2019; Zhang et al., 2018).

The study by VanKuiken et al. (2017) showed that students who benefited from an in-person very short intervention in class found the experience calming, allowing them to focus, set aside distractions, and feel a sense of community, although these findings were exclusively based on qualitative evaluations. Nevertheless, the lack of a control group made it impossible to discern if the results were due to the intervention or to other variables. Taylor et al. (2020) compared the experimental group with a control group and found significant improvements in self-compassion and coping self-efficacy. However, the authors noted that their findings should be replicated in larger samples, since the generalizability of their results was limited by the small sample size (*n* = 61), which also limited the statistical power.

There are potential advantages, as well as disadvantages, in digital mindfulness training, as rightly noticed by Mrazrek et al. (2019). Among the advantages there is the reduction of geographical and logistical constraints and greater accessibility (Asuncion et al., 2010; Bernard et al., 2004; Fichten et al., 2000). At the same time, according to the review of Mrazek et al. (2019), online MBIs had quite low rates of completion and average attrition rate resulted around 35%; even with those participants who completed the intervention, nearly half of the reviewed studies mentioned problems with adherence, with participants not fully engaging in the program of practices and lessons as prescribed.

As highlighted by Mrazek et al. (2019), an online MBI should take into account the specific motivations and needs of the students attending the class, review regularly the proposed content and the assigned homework, and rely more on visuals and graphics audio and books, as in the interventions by Bennett et al. (2018) and Taylor et al. (2020). Moreover, it is important that anyone offering a MBI should master the basic concepts underlying mindfulness, embody the skills, and have personal practice. In particular, high levels of treatment fidelity are associated with stronger program effects (Borrelli, 2011).

A strength of this study is having monitored the degree of students’ engagement in homework practice and if their adherence influenced the outcomes of the intervention. Results showed that the adherence to the daily mindfulness practice modulated the increase of academic self-efficacy, self-regulation, and fulfilment of needs scores from pre- to post-intervention. Therefore, meditating on a daily basis improved the capability of self-regulating bodily states and the perception of the self as self-efficacious, and of the attended class as a source of personal satisfaction. It is likely that a virtuous circle set out. Adherence contributed to increase self-efficacy, self-regulation, and fulfilment of needs, and, in turn, these latter positively affected adherence to practice. It is also notable that many students kept practicing formal mindfulness meditation throughout the course. In this regard, it is possible that the mindfulness instructor had been capable to motivate students in such a way that mindfulness practice represented for them not an academic duty, but an opportunity of personal and academic growth. Future studies could better explore the variables affecting students’ adherence to the mindfulness practice.

Our study presents several limitations that should be acknowledged. First, we did not employ a fully randomized design which entails random assignment of participants to either the experimental or the control group, but we resorted to cluster randomization, albeit with a large sample of participants. In the type of intervention study reported here, only a randomized controlled trial (RCT) could demonstrate that the observed benefits are due to the employed MBI, and not to other variables. Second, we only used self-reported measures, which could lead to common methods bias: the use of multiple-item rating scales within the same survey could produce spurious effects due to the measurement instruments rather than to the constructs that are being measured. As a result, it is possible to find spurious correlations among the items measuring these different constructs caused by, e.g., priming effects and social desirability, regardless of the actual correlations among the measured constructs (Podsakoff et al., 2012). Future studies could include multiple informants and types of assessment methods.

Third, future RCTs could entail a follow-up period to assess the stability of the observed improvements, and provide more robust evidence of the causal relation between the intervention and its outcomes. Fourth, the intervention was not compared to an active control condition. This means that the observed improvements in the experimental group may be attributed to participating in a practical activity within the course, rather than to the mindfulness intervention specifically. Future studies could include an active control condition, such as a leisure activity.

However, despite these limitations, the large sample size and the presence of a control group allow to draw reliable conclusions from our data: we delivered a brief online MBI within regular class time in a large sample, and our research design included both an experimental and a control group. Moreover, our protocol included, besides the mindfulness practice during regular class hours, homework assignments, namely the daily breathing meditation, which could enhance an internalization of the practice and its use in the daily routine, increasing the odds of enduring effects of the intervention. In summary, this study offers initial evidence on the effectiveness of brief online MBIs in enhancing students’ attention resources, academic self-efficacy, and sense of community. Integrating these practices into college courses could be a very accessible way of incorporating mindfulness into college life and could help improve students’ participation and overall academic success.

## Data Availability

The datasets generated and analyzed during the current study are available from the first author on reasonable request.
